# A Self-Adaptive Behavior-Aware Recruitment Scheme for Participatory Sensing

**DOI:** 10.3390/s150923361

**Published:** 2015-09-16

**Authors:** Yuanyuan Zeng, Deshi Li

**Affiliations:** 1 School of Electronic Information, Wuhan University, Wuhan 430072, China; 2 Collaborative Innovation Center for Geospatial Technology, Wuhan 430072, China; E-Mail: dsli@whu.edu.cn

**Keywords:** participatory sensing, data recruitment, self-adaptive, behavior-aware

## Abstract

Participatory sensing services utilizing the abundant social participants with sensor-enabled handheld smart device resources are gaining high interest nowadays. One of the challenges faced is the recruitment of participants by fully utilizing their daily activity behavior with self-adaptiveness toward the realistic application scenarios. In the paper, we propose a self-adaptive behavior-aware recruitment scheme for participatory sensing. People are assumed to join the sensing tasks along with their daily activity without pre-defined ground truth or any instructions. The scheme is proposed to model the tempo-spatial behavior and data quality rating to select participants for participatory sensing campaign. Based on this, the recruitment is formulated as a linear programming problem by considering tempo-spatial coverage, data quality, and budget. The scheme enables one to check and adjust the recruitment strategy adaptively according to application scenarios. The evaluations show that our scheme provides efficient sensing performance as stability, low-cost, tempo-spatial correlation and self-adaptiveness.

## 1. Introduction

Participatory sensing [1,2] is a novel and promising sensing paradigm with the fast development of mobile smart devices. The participatory sensing paradigm enables people with smart devices as the social sensors to be part of the sensing campaign to collect data from the ambient environment. The sensing data can then be shared and analyzed to reveal a certain pattern of the city. Participatory sensing is well-suited for applications such as air quality monitoring, noise monitoring, and transportation monitoring, *etc*. The objective of participatory sensing is to supervise the process and results of social activities. According to this, the recruitment scheme of social participants with self-adaptive data quality within varied realistic scenario becomes the key problem in participatory sensing. The participants are engaged in their own daily activities when they take part in the sensing campaign. Thus, the sensing activity is restrained by the daily behavior. The behavior then results in the data quality contributed by recruited participants. For a participatory sensing campaign to succeed, the participant recruitment has to stay connected with the living, working, and entertaining behavior.

In the paper, we present a self-adaptive behavior-aware recruitment (SBR) scheme for participatory sensing that recruits the well-suited participants for sensing tasks according to the behavior of participants. The recruitment scheme is self-adaptive to the sensing data quality, *i.e.*, the recruit strategy will change dynamically according to the data quality of current status.

The main contributions of this work can be summarized as follows:

(1) SBR scheme is proposed to recruit participants according to tempo-spatial-correlated behavior and valid data quality to provide efficient data collection in participatory sensing.
(2) SBR enables a self-adaptive recruitment strategy to obtain a relatively stable sensing performance according to varied application scenarios.
(3) SBR is designed for the realistic social participatory sensing scenario when people are recruited as they commit to their daily activity at the same time. The SBR scheme does not need pre-defined ground truth or any instructions on where and when to sample during the participatory sensing campaign.

The rest of this paper is organized as follows. Section 2 presents the related work. Section 3 outlines the recruitment framework. We present the SBR scheme in Section 4. Section 5 is performance evaluation. Finally, Section 6 concludes this paper.

## 2. Related Work

Participatory sensing has become one of the most promising application paradigms for human-centric mobile sensing. The following provides a brief overview of related work in terms of participatory sensing paradigm and data recruitment, respectively.

The state-of-the-art literature [3] shows that future mobile sensing systems develop toward the people-centered and environment-centered sensing paradigm. The sensing domain can be home, urban, vehicular, *etc*. As the concept of participatory sensing was proposed, lots of research on participatory sensing paradigms was brought forward. Common Sense [4] is a participatory sensing system based on mobile handheld devices, which enables individuals and social community to be a part of air quality monitoring and measurement for an urban city environment. Ear-phone [5] is an open participatory sensing platform for urban noise surveillance by using Nokia N95 and HP iPAQ to monitor the noise data, which is a better option for noise monitoring with moderate price and timeliness. Hasenfratz *et al.* [6] proposed participatory air pollution monitoring with both built-in sensors of smartphones and USB plug-in sensors for data sensing. Zhou *et al.* [7] proposed a participatory sensing paradigm that can predict bus arrival time with mobile phone.

Predic *et al.* [8] proposed the ExposureSense participatory sensing framework with general information infrastructure based on smartphones, virtual sensors, and deployed wireless sensor networks through web services. Sun *et al.* [9] proposed a participatory sensing system for air quality monitoring based on static wireless sensors and smartphones held by people.

The preliminary data recruitment in participatory sensing is based on simple schemes: random selection of data contributors and a naive scheme that asks all the contributors in the area of interest to contribute data. Although they are easy to be implemented, the schemes have potentially serious disadvantages, such as sensing coverage, redundancy, and cost. The state-of-the-art data recruitment schemes focus on the recruitment of suitable contributors as participants with good performance oriented toward the sensing campaign requirement. According to the metrics during recruitment, the schemes include three main categories.

The first one is coverage-based recruitment. The tempo-spatial coverage is the first consideration for most of the participatory sensing campaign. Estrin *et al.* [10] proposed recruitment schemes to choose participants for data collection based on geographic and temporal availability with consideration of participation habits. Tuncay *et al.* [11] proposed an autonomous distributed recruitment and data collection framework for opportunistic sensing. They proposed a fully-distributed, opportunistic sensing framework to recruit the participants within the coverage of sensing missions, which is based on data mining of the human historical mobile trajectories. Hamid *et al.* [12] proposed an efficient data recruitment scheme for urban sensing based on vehicle trajectories. They analyzed the sensing coverage according to the mobile trajectories of vehicles and then recruited the suitable vehicles for urban sensing. The above schemes are mostly based on the mobility analysis according to the historical trajectories, and then choose the suitable participants with tempo-spatial coverage.

The second category is reputation-based recruitment. The reputation-based recruitment schemes are mainly based on metrics for cross-campaign and campaign-specific situations. For campaign-specific participatory sensing, the metrics usually include timeliness, relevance of data, participant willingness, and degree of trustworthiness of data. For cross-campaign participatory sensing, the metrics also include the number of previous campaigns taken, the experience of participants, and fairness, *etc*. In [10], they also proposed a reputation-based recruitment scheme, which proposed to use Beta distribution to build the reputation metric and then calculate the probability of participation. Amintoosi *et al.* [13] proposed to choose trustworthy participants among friends and friends’ friends of online social networks. They [14] then proposed a novel recruitment scheme that considers both the quality of contribution and the trustworthiness level of participant within the social networks combined via a fuzzy inference system to arrive at a final trust rating for a contributor. Hamid *et al.* [15] proposed the reputation assessment and pricing model for the optimal reputation-aware recruitment framework to calculate the reputation score and recruitment cost. They proposed to recruit vehicles for public sensing considering the spatio-temporal coverage of participants along with the participant reputation within a budget limit.

The third category is expertise-based recruitment. The expertise-based recruitment schemes choose the well-suited participant with relevant expertise or experience for a given topic in a sensing campaign. Expert finding problems have been excessively studied in social networks [16,17]. In [16], they proposed a propagation-based approach for expert finding in social networks. In [17], they proposed a Bayesian hierarchical model for expert finding considering both social relationships and contents. Wang *et al.* [18] proposed a participant recruitment framework for crowdsourcing system, which identify the well-suited participants with particular kind of domain knowledge based on clustering in particular spatio-temporal spaces.

Beyond the above categories, there are some other recruitment schemes to identify the participants according to more than one metric. In fact, it is usually in the form of a hybrid scheme with some combined metrics [13,15,19] from the above categories for better recruitment performance in participatory sensing campaign.

Some of the research [20,21,22] shows that human mobility in daily activities obeys a certain pattern with strong tempo-spatial correlation and periodic discipline. Different from the above schemes, our recruitment scheme considers the behavior of participants during the daily activities as an important factor to identify the data quality with metrics combines coverage, reputation and expertise in participatory sensing. The scheme is also self-adaptive toward the change of participant behavior during daily activity as well as sensing activity.

## 3. Behavior Modeling in Participatory Sensing

The behavior modeling includes the modeling of tempo-spatial-related behavior and behavior ties.

### 3.1. Tempo-Spatial-Related Behavior

**Figure 1:**
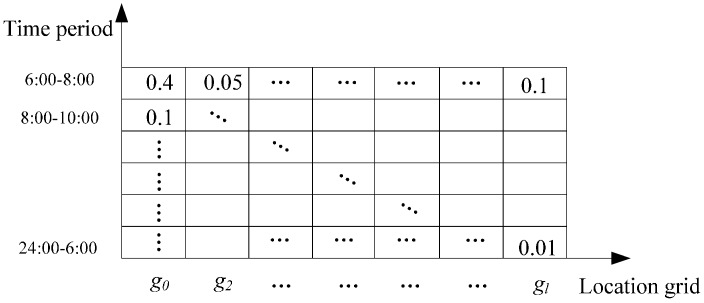
The association matrix based on mobile trajectory.

We represent the mobile trajectory of people held with smartphones or the other smart devices in participatory sensing using the association matrix as illustrated in Figure 1. In the matrix, the row corresponds to a location grid. The location grid *g*_0_, *g*_2_, …, *g_l_* is originated from the place division of the interested area for sensing task. The column corresponds the time period at the location grid for the typical time duration, such as a day or a week. In people’s daily life, behavior varies within different time periods. The value of column is based on this; e.g., time 24:00–6:00 is the rare moving time duration for most of the people in daily life. The other time durations like 6:00–8:00 and 8:00–10:00 show more activity of moving trajectory. The matrix elements represent the percentage of time that people stay inside the grid.

We utilize the method in [23] to capture the eigenbehavior vector for a given participant. For participant *x* with association matrix *A*, the singular value decomposition (SVD) [24] is applied as in Equation (1):

(1) $$A=U\cdot \sum \cdot V^{T}$$

In which, a set of eigenbehavior vectors representing the leading trends within the typical time period can be obtained from the column of *U*; *i.e.*, the rows of matrix *V^T^*.∑ is the diagonal matrix with corresponding singular values, *σ*_1_, *σ*_2_, …, *σ*_rank_(*A*). The weight of each eigenbehavior vectors can be achieved as in Equation (2):

(2) $$w_{i}=\frac{\sigma_{i}^{2}}{\sum_{j=1}^{rank(A)}\sigma_{j}^{2}}$$

The tempo-spatial similar behavior of two participants can be achieved through the cosine similarity of eigenbehavior vectors. For user *x* and *y* with association matrix *A* and *A’*, the similarity is calculated as shown in (3):

(3) $$sim(x,y)=sim(U_{x},U_{y})=\sum_{i=1}^{rank(U_{x})}\sum_{j=1}^{rank(U_{y})}w_{xi}w_{yi}|U_{xi}\cdot U_{yj}|$$

### 3.2. Behavior Tie

The behavior in daily life of people is constrained by the social activities and affected by social relations. The user behavior tends to be with homophile, which explains the reason for building ties among similar users. There are strong ties and weak ties in social life that affect the substantial social participatory sensing campaign, *i.e.*, the ties affect participation.

We use information theory-based methods to measure the correlation of participation to the probability of happening. The mutual information [25] metric measures the dependence of two users in terms of their participation behavior in participatory sensing campaigns. Given two random participating events *X* and *Y* with marginal probability mass function *p*(*x*) and *p*(*y*), representing the probability of the participation for user *x* and user *y* in a specific participatory sensing campaign, and the joint probability mass function of *p*(*x*,*y*),representing the joint probability of the participation for user *x* and *y*, their mutual information *I*(*X*;*Y*) is defined as the relative entropy between the joint distribution and their product distribution as in Equation (4). In which, the mutual information function *I*(*X*,*Y*) quantifies the amount of information (in units of bits)that can be obtained from user *x* about another user *y* participants in a specific sensing task:

(4) $$I(X;Y)=\sum_{x\in X,y\in Y}p(x,y)\log\frac{p(x,y)}{p(x)p(y)}$$

The normalized mutual information (NMI) represents the normalized behavior correlation of two users in participatory sensing, *i.e.*, behavior tie in participatory sensing. The NMI can be defined as in Equation (5), where *H*(*X*) and *H*(*Y*) are the entropy of *x* and *y*. The NMI value (*i.e.*, behavior tie as we defined) is between 0 and 1.The value 1 implies *X* and *Y* with the same participating behavior in the sensing campaign, and value 0 implies *X* and *Y* with the opposite participating behavior:

(5) $$NMI(X;Y)=\frac{I(X;Y)}{\sqrt{H(X)H(Y)}}$$

According to the historical record of participants for a certain long time duration *T*, the user *x* moves among grid *g*_0_ to *g_l_* with *t_x_* as the time fraction of participation. The user *y* moves among grid *g*_0_ to *g_l_* with *t_y_* as the time fraction of participation. The entropy *H*(*X*) and *H*(*Y*) is calculated as shown in Equation (6):

(6) $$\begin{array}{l} H(X)=-\sum_{g_{0}}^{g_{l}}\frac{t_{x}}{T}\log\frac{t_{x}}{T}\\ H(Y)=-\sum_{g_{0}}^{g_{l}}\frac{t_{y}}{T}\log\frac{t_{x}}{T} \end{array}$$

Based on this, we have Equations (7) and (8):

(7) $$I(X;Y)=\sum_{g_{0}}^{g_{l}}\frac{t_{x,y}}{T}\log\frac{\frac{t_{x,y}}{T}}{\frac{t_{x}}{T}\frac{t_{y}}{T}}$$

(8) $$NMI(X;Y)=\frac{\sum_{g_{0}}^{g_{l}}\frac{t_{x,y}}{T}\log\frac{\frac{t_{x,y}}{T}}{\frac{t_{x}}{T}\frac{t_{y}}{T}}}{\sqrt{\sum_{g_{0}}^{g_{l}}\frac{t_{x}}{T}\log\frac{t_{x}}{T}\sum_{g_{0}}^{g_{l}}\frac{t_{y}}{T}\log\frac{t_{x}}{T}}}$$

## 4. SBR Scheme

We assume that all users with handheld smart devices can get to know sensing tasks from a sensing task distribution platform by service providers. The users that join the platform and agree to be recruited can become a participant. The recruitment is executed during users’ daily life without intervening in their daily activities.

### 4.1. Recruitment Recommendation

Data quality is a key factor to decide the recruitment in participatory sensing. From the historical sensing data, some metrics such as data accuracy, redundancy, relevance, completeness, and timeliness can be considered to measure the data quality contributed, which can assist to build the participants’ reputation. A final participant data quality rating score can be rated by the service provider and then be computed based on the weighted average metric value as shown in Equation (9), in which the metrics can be decided and chosen by the service provider according to sensing requirements:

(9) $$R=\alpha_{1}\cdot accuracy+\alpha_{2}\cdot timeliness+\alpha_{3}\cdot relevance+\alpha_{4}\cdot completeness+\alpha_{\text{5}}\cdot timelinness$$

Considering data quality is highly related with user behavior in sensing activities along with their daily life, we use recommendations in data quality prediction. Based on the behavior tie for users in participatory sensing, we can predict the unknown user behavior of participation by considering the user interactions. The data quality based on recruitment recommendation becomes a collaborative filtering problem. The behavior tie implies the correlation of user behavior in participatory sensing with the value between 0 and 1. Then we choose the users with correlated behavior to make recommendation for a specific sensing campaign *i*, *i.e.*, with *NMI*(*X_i_*;*Y_i_*) ≥ *NMI*_th_. For user *x* that satisfies the above requirement, the recommendation with a weighted policy is shown as in Equation (10), in which we get the recommended data quality rating score as *PR_y,i_* with participation candidate *y* toward a specific sensing campaign *i*.

(10) $$PR_{y,i}=\bar{R_{y}}+\frac{\sum NMI(X_{i},Y_{i})\cdot (R_{x,i}-\bar{R_{x}})}{\sum \left|NMI(X_{i},Y_{i})\right|}$$

### 4.2. Recruitment Programming

The recruitment metrics that we consider in the scheme include tempo-spatial behavior characteristics of participants, data quality that can be achieved through the recruitment strategy, and budget that users can afford. Based on this, the recruitment problem can be modeled as a linear programming optimization problem for recruitment objectives. For a specific participatory sensing task *i*∈*I* with the participants set P* = {*p*_1_, *p*_2_, *…*, *p_n_* }, the recruitment programming can be formulated as shown in Equations (11–14):

(11) $$\max\;\sum_{k=1}^{n}sim(p_{i},A_{std,i})$$

subject to:

(12) $$sim(p_{ki},A_{std,i})\geq sim_{th}\;\forall p_{k}\in P^{*}$$

(13) $$PR_{p_{k},i}\geq R_{th}\;\forall p_{k}\in P^{*}$$

(14) $$\sum_{k=1}^{n}budget\;(p_{k})\leq bd_{th}$$

In Equation (11), *AM_std,i_* is the typical tempo-spatial association matrix that can be given by the upper-layer application or through a long-time observation for sensing activities with the required coverage of grids and time fractions. *sim*(*p_i_*,*AM_std,i_*) is the similarity for partition candidate *p_i_* with the required tempo-spatial coverage. We have *sim*(*p_i_*,*A_std,i_*) = *sim*(*A_pi_*,*A_std,i_*), where the similarity value is between 0 and 1, in which 0 implies no similarity with the required tempo-spatial characteristics and 1 implies the exact required tempo-spatial characteristics. The objective of recruitment programming is to maximize the total tempo-spatial behavior similarity for participants, when it subjects to Equations (12)–(14). In Equation (12), *sim_th_* is the similarity threshold required for a sensing task, which provides the required tempo-spatial behavior similarity for participants. In Equation (13), *R_th_* is the threshold for data quality rating required for sensing task *i*. It provides the constraint of data quality. In Equation (14), *bd_th_* is the budget threshold that users can afford. It provides the budget constraint in sensing task, e.g., battery energy *etc*. The above threshold values are given and adapted by upper-layer applications.

### 4.3. Self-Adaptive Strategy

Sensing data is the main concern for participatory sensing campaign. Data quality can reflect the effectiveness of recruitment. However, the recruitment performance with participants will change with varied people’s behavior in daily life. We utilize a self-adaptive recruitment strategy to update the participants according to the dynamic scenarios. The self-adaptive scheme is to check the data quality periodically with interval parameter *T_d_*. If the data quality for current participant *p_k_*∈P* cannot satisfy the requirement for a specific check duration, *i.e.*, *R_pk_* < *R_th_*, the unqualified participant will be removed. If the overall data quality for current participant set P* cannot satisfy the requirement for a specific check duration, *i.e*., R_P*_ < *R_th_*, a new round of recruitment is involved to recruit new participants to provide the valid data quality.

## 5. Evaluation

**Figure 2:**
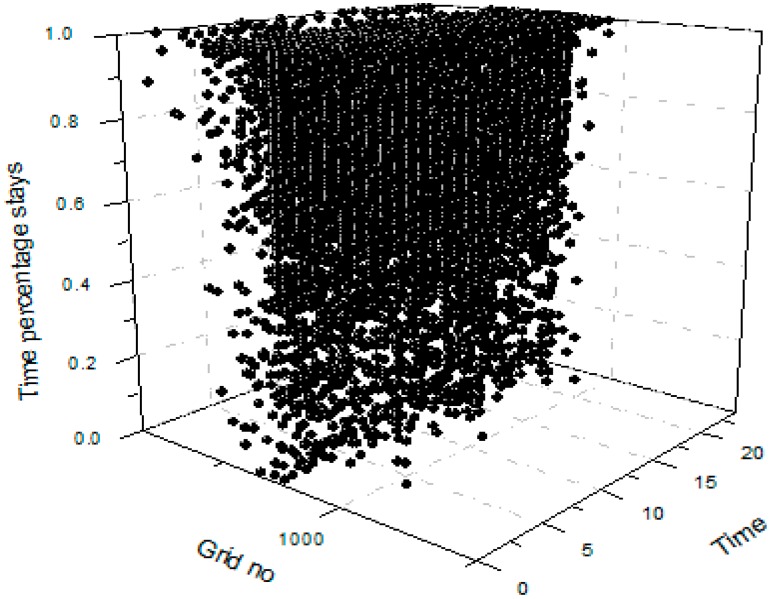
The tensor illustration of Geolife user data.

We implemented the proposed scheme by programming in Java to make performance evaluations. Our evaluation is based on real trajectory sets of people’s daily life by using the Geolife dataset [26], which was a project of Microsoft Research Asia with 182 users over five years from April 2007 to August 2012. Through data analysis, we divide the trajectory area of Geolife into 5874 grids according to the latitude and longitude range of the trajectory. On the other hand, we divide the trajectory data into each hour according to the time within a day. Based on that, we extract the association matrix data for each user with grid number, hour number, and stay duration within the specific grid and hour. The data of 182 users in the Geolife dataset forms a tensor that can be illustrated as shown in Figure 2.

The following section evaluates the scheme according to stability, low-cost, tempo-spatial correlation, and self-adaptiveness. Since real experiments in participatory sensing are not easy to organize, most of the research work conducts simulations to make evaluations. To our knowledge, current simulations for participatory sensing are based on different scenarios with specific assumptions and controlled environments. There are different moving models and social groups set for participants who contribute data to sensing campaigns; e.g., some of the work assumes there are experts with the ground truth route to guide people moving for sensing. Some of the work assumes to use predefined random moving models and friendship relation models. The above leads to different metrics used to evaluate and observe the impact on the system performance. The performance metrics we considered here include number of participants and average stay duration fraction. The number of participants metric shows the recruitment cost with a certain sensing coverage and reputation as considered in SBR. The average stay duration fraction metric shows the recruitment effectiveness for the current strategy. Since people only join the sensing campaign within their daily life, the trajectory is dynamic. The instable participants may join and then leave the sensing campaign with only biased data and cannot provide a sustained sensing. The bigger average stay duration fraction for the specific location (grid) shows that people with more stable sensing habits probably provide more reliability from a sustained contributor. As in [10], our scheme is compared with three recruitment schemes: random, naïve, and greedy. The random scheme selects participants randomly. The naive scheme selects participants that cover the grids and time without considering what existing selected participants covered. The greedy scheme selects participants that cover the most grids and time with considering what existing selected participants covered. We generate the target scenarios of participatory sensing campaign by choosing the random grids, random hours, and random stay durations within the most people’s activity area and time period. Our evaluation results are based on the average of five groups of different random scenarios.

### 5.1. Recruitment for a New Participatory Sensing Campaign

**Figure 3:**
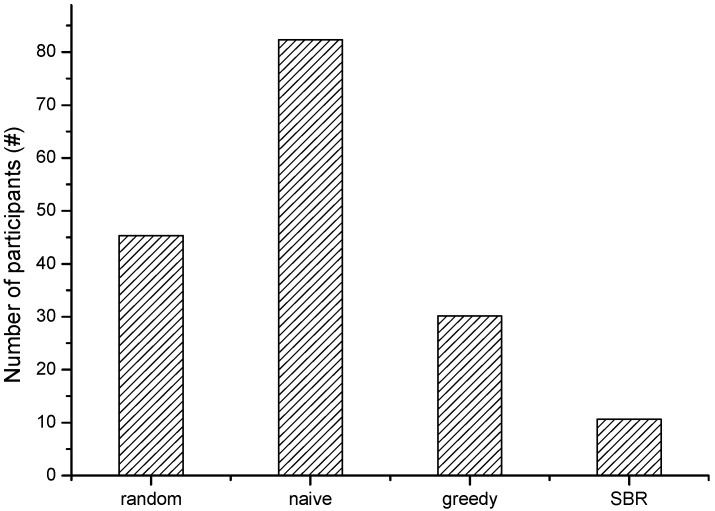
The number of participants with different recruitment schemes.

**Figure 4:**
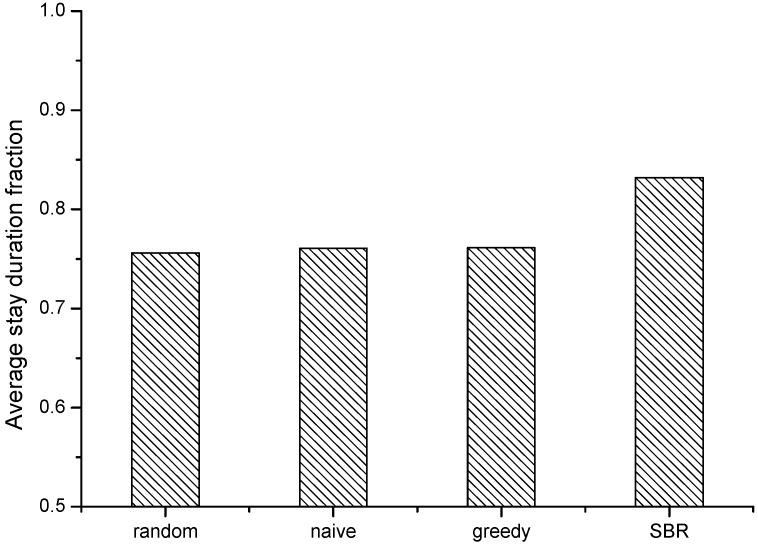
The average stay duration with different recruitment schemes.

For a new participatory sensing campaign, the participant recruitment is based on the history trajectory. We use the Geolife data before year 2009 as the history trajectory. In a new sensing task, the data quality rating encounters the cold start. We assume all participant candidates have the same initial data quality score that is qualified. We also assume that we can afford the budget during the evaluation. Figure 3 shows the number of recruited participants with our scheme SBR when compared with the other three schemes. It implies SBR is less costly (*i.e.*, needs less to pay for the participants within the sensing requirements).From the result, we can see that SBR recruits the least number of participants for the target sensing scenario. The naïve scheme recruits the most number of participants, because it only selects participants with the valid tempo-spatial coverage for the task scenario without considering the existing participants. The greedy scheme reduces the number of participants by considering the existing participants’ coverage. The random scheme still recruits considerable participants by arbitrary selection. Figure 4 shows the average stay duration time fraction within the task grids of the recruited participants (named average stay duration fraction) by using different recruitment schemes. The average stay duration implies the stability of participants for the sensing task, *i.e.*, the longer the average stay duration, it means the participant stays longer in the expected grids within the expected time for the sensing task. It shows that people provide sensing data with more stable sensing habits and help to improve the data quality. The results show SBR achieves the best average stay duration. This implies that SBR recruits participants with relatively good stability of tempo-spatial correlation for the sensing task. The other three schemes are with less average stay duration, and they have slight differences among one another.

**Figure 5:**
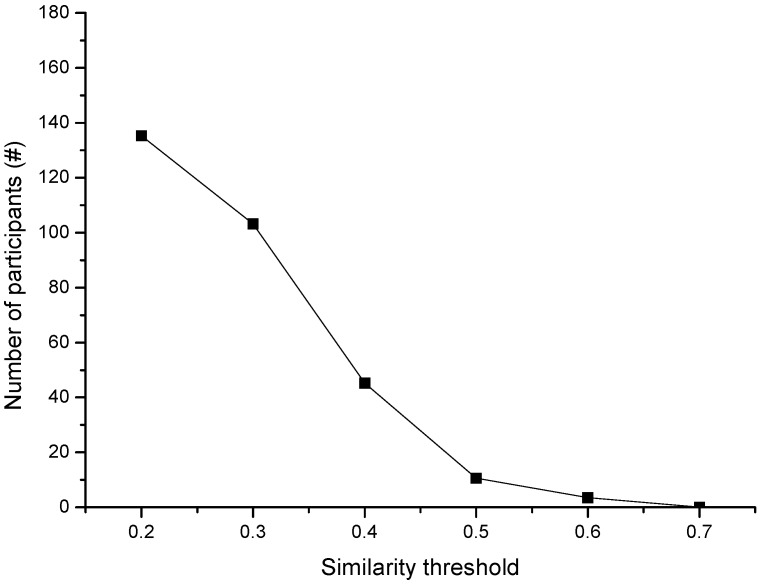
The number of participants with different similarity threshold.

**Figure 6:**
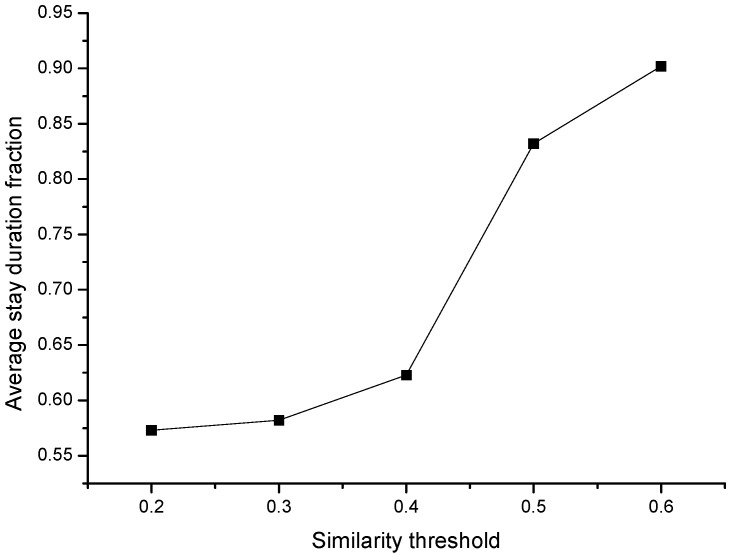
The average stay duration with different similarity threshold.

We then made evaluations with a different similarity threshold to see the effect of tempo-spatial correlation within a different threshold selection. Figure 5 shows the participant number when we choose a different similarity threshold *sim_th_*. The results show the participant number decreases as the similarity threshold increases, but the unreasonable similarity threshold incurs too many or too few participants. Too many participants increase the cost and incur the possible tempo-spatial redundancy, and too few participants cannot satisfy the tempo-spatial coverage. Figure 6 shows the average stay duration when choosing different similarity threshold *sim_th_*. The results show the average stay duration increases as the similarity threshold increases. As we set threshold *sim_th_* ≥ 0.5, the average stay duration gradually arrives at a considerable value than *sim_th_* < 0.5. This implies that the tempo-spatial-related sensing habits of people tend to be more valuable for sensing tasks when choosing *sim_th_* ≥ 0.5.

### 5.2. Recruitment for a Sustained Participatory Sensing Campaign

Based on the last subsection, we further consider the sensing campaign within sustained sensing campaign scenarios. We use the Geolife data from the 1st to 10th month in year 2009 as the assumed current on-time updated trajectory (considering the sparse trajectory after October 2009 that may incur poor coverage, we choose data of the 1st to 10th month in 2009). The update interval is taken as one month. The data quality is recommended after a random rating during the participatory sensing. In realistic situations, the data quality can be rated according to some combined metrics, such as data accuracy, redundancy, and timeliness, *etc.* In sustained sensing campaign scenarios, we can predict and decide the recruitment based on certain sensing related pattern according to the historical data, since human daily activity shows a certain tempo-spatial correlations. On the other hand, as time goes by, the application scenario varies. The behavior changes as users leave and new users join in the sensing tasks. However, we can potentially still find some relatively fixed patterns from people’s daily activity and design an adaptive strategy through the analysis of the historical data. Due to this, the self-adaptiveness of SBR can handle the varied scenarios by adapting the recruitment strategy.

**Figure 7:**
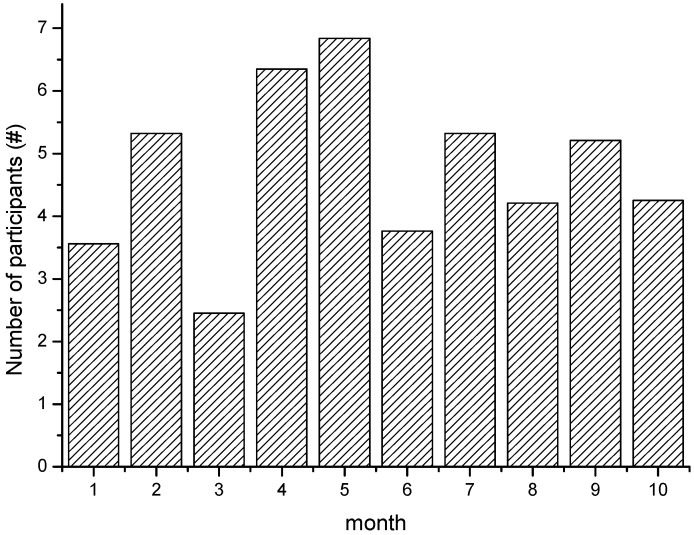
The self-adaptive participant number.

**Figure 8:**
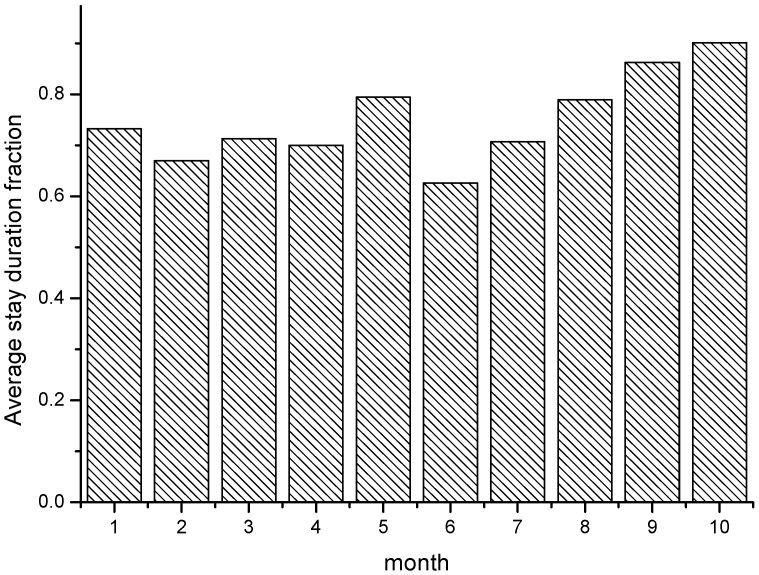
The self-adaptive average stay duration.

Figure 7 shows the self-adaptive participant number that changes with the update interval, *i.e.*, months. At the end of each update interval, a data quality-based check is held to see if current data quality is qualified and needs to trigger the next new recruitment. The results show SBR can self-adapt the strategy to recruit a suited number of participants that satisfy the current application scenarios. Figure 8 shows the self-adaptive average stay duration, which can stay at a certain good level through self-adaptiveness. From the results, we can see the recruitment provides self-adaptiveness with a relatively stable sensing performance as the participant trajectory changes. This shows SBR can adjust the recruitment strategy and provide an efficient sensing capability within varied application scenarios.

## 6. Conclusions

In this paper, we propose a self-adaptive behavior-aware recruitment scheme for participatory sensing considering tempo-spatial behavior and data quality incurred. The scheme utilizes historical trajectory analysis to model behavior similarity, based on tempo-spatial correlation, and behavior tie, based on NMI. We then propose a data quality assessment based on collaborative filtering. Based on this, we formulate the recruitment scheme as a linear programming optimization problem by combining coverage, data quality, and budget. The scheme enables one to adapt a recruitment strategy according to varied application scenarios. Our evaluation is based on a realistic mobile trajectory dataset without a pre-defined ground truth or instruction for when and where to sample, which is suitable for realistic social scenario in participatory sensing. Our scheme provides good stability, low-cost, tempo-spatial correlation and self-adaptiveness for participatory sensing campaigns. Our future work includes the implementing of SBR into our campus participatory sensing test bed.
